# Effects of modified‐constraint induced movement therapy based telerehabilitation on upper extremity motor functions in stroke patients

**DOI:** 10.1002/brb3.3569

**Published:** 2024-06-14

**Authors:** Fettah Saygili, Arzu Guclu‐Gunduz, Sefa Eldemir, Kader Eldemir, Cagla Ozkul, Gorkem Tutal Gursoy

**Affiliations:** ^1^ Faculty of Health Sciences, Department of Physiotherapy and Rehabilitation Aydın Adnan Menderes University Aydın Türkiye; ^2^ Faculty of Health Sciences, Department of Physiotherapy and Rehabilitation Gazi University Ankara Türkiye; ^3^ Faculty of Health Sciences, Department of Physiotherapy and Rehabilitation Sivas Cumhuriyet University Sivas Türkiye; ^4^ Faculty of Health Sciences, Department of Physiotherapy and Rehabilitation Ordu University Ordu Türkiye; ^5^ Department of Neurology, Health Ministry of Turkish Republic Ankara Bilkent City Hospital Ankara Türkiye

**Keywords:** modified‐constraint induced movement therapy, stroke, telerehabilitation, upper extremity

## Abstract

**Introduction:**

The aim of this study is to investigate the effects of Modified‐Constraint Induced Movement Therapy (m‐CIMT) based telerehabilitation on upper extremity motor functions in stroke patients.

**Methods:**

Eighteen stroke patients were included and randomly allocated into two groups. The Tele‐CIMT (modified‐constraint induced movement therapy‐based telerehabilitation) (*n* = 10) group received m‐CIMT based telerehabilitation for 90 min a day, 5 weekdays for 3 weeks at home. Additionally, both the Tele‐CIMT group and the control group (CG) (*n* = 8) underwent the home exercise program aimed at improving range of motion, active movement, balance, and walking every weekday for 3 weeks at home. The outcome measures were the Stroke Rehabilitation Assessment of Movement Scale (STREAM), Fugl‐Meyer Upper Extremity Motor Evaluation Scale (FM‐UE), Wolf Motor Function Test (WMFT), 9‐Hole Peg Test (9‐HPT), grip strengths, pinch strengths, Motor Activity Log‐28 (MAL‐28), and Functional Independence Measure (FIM).

**Results:**

Significant group‐by‐time interactions on STREAM, FM‐UE, WMFT, grip strength, pinch strengths, MAL‐28, and FIM were found to be in favor of the Tele‐CIMT group. Additionally, post hoc analyses revealed that the Tele‐CIMT group significantly improved in terms of these parameters (*p* > .05).

**Conclusion:**

This is the first randomized controlled trial showing that Tele‐CIMT improved upper extremity motor functions and activities of daily living in stroke patients. Tele‐CIMT can help improve the upper extremities in stroke survivors who have difficulties reaching rehabilitation clinics.

## Graphical abstract

Investigating the effects of Modified‐Constraint Induced Movement Therapy (m‐CIMT) based telerehabilitation on upper extremity motor functions in stroke patients.

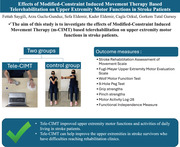

## 1 INTRODUCTION

Stroke, which causes the death of 6.5 million people in the world every year, is described as a syndrome characterized by rapid settlement of signs and symptoms of focal loss of cerebral function without being caused by other vascular causes (Feigin et al., 2017; World Health Organization, 2000). After a stroke, motor disorders lead to functional deficiencies in the upper extremity (UE), which result in inadequacies in the activities of daily living (ADLs) and limit the individual's return to their social and professional lives (Kissela et al., 2012).

As known, it is more difficult to regain functional ability in the UEs after stroke and functional recovery can be achieved in only 50% of patients (Broeks et al., 1999; Kwakkel et al., 1999). One of the most important reasons for that is the affected UE is used less than the lower extremity, both in rehabilitation and in daily life after a stroke. At this point, it is recommended to use interventions, such as constraint‐induced movement therapy (CIMT), in which the extremity is exposed to intensive practice, should be used (Abdullahi et al., 2023). In CIMT, which is one of the methods with the highest evidence value in UE rehabilitation after stroke (Shi et al., 2011; Sirtori et al., 2009), the less affected UE of the participants is restricted with a mitt 90% of the time while they are awake. At the same time, the participants participate in 6‐h of daily activity sessions for 10–15 consecutive days (Taub, 2000). However, CIMT can be a difficult method to apply due to the long duration of restraint with a mitt and daily treatment time. Therefore, in order to increase the participation of patients, Page et al. (2001) developed the modified‐CIMT (m‐CIMT) protocol by applying the restriction period in the original protocol to 5 h a day and the activity sessions to 30 min a day, 3 days a week for 3 weeks.

Obviously, after discharge, many patients cannot apply to rehabilitation centers due to lack of transportation, economic inadequacies, and so on, and are deprived of treatment. In this case, the most commonly used method of providing rehabilitation services to patients is telerehabilitation (Johansson & Wild, 2011). However, although the popularity of telerehabilitation has increased, especially with the COVID‐19 pandemic, there are limited studies on the effectiveness of CIMT and m‐CIMT based telerehabilitation. To the best of our knowledge, there are four studies in which CIMT and m‐CIMT were applied via telerehabilitation. Original CIMT was applied in two of them (Pickett et al., 2007; Uswatte et al., 2021), and m‐CIMT was applied in the other two (Page & Levine, 2007; Smith & Tomita, 2020).

When these limited number of studies in which CIMT or m‐CIMT was applied via telerehabilitation were evaluated collectively, in two of them, it was found that the number of cases was insufficient, and most of them did not include comprehensive outcome measures related to UE functional skills (Page & Levine, 2007; Pickett et al., 2007; Smith & Tomita, 2020; Uswatte et al., 2021). Additionally, two of these articles are case series (Page & Levine, 2007; Pickett et al., 2007), one is a preexperimental design study (Smith & Tomita, 2020) and the other is a randomized controlled proof of concept study (Uswatte et al., 2021) that evaluated only the amount of use of UE. As a result, there is no randomized controlled study investigating the effects of m‐CIMT based telerehabilitation (modified‐constraint induced movement therapy‐based telerehabilitation [Tele‐CIMT]) on UE motor functions and ADLs has been found. Based on this, the aim of our study is to investigate the effectiveness of Tele‐CIMT on UE motor functions and ADLs in stroke patients.

## 2 METHODS

### 2.1 Participants

Eighteen stroke patients who were diagnosed with stroke by a specialist neurologist in the Ankara Bilkent City Hospital Neurology Outpatient Clinic and referred to the Gazi University Department of Physiotherapy and Rehabilitation, neurorehabilitation outpatient clinic, were included in the study. Inclusion criteria for the study were (Kunkel et al., 1999; Page et al., 2001, 2002, 2005; Taub et al., 1993): (1) being diagnosed with stroke for the first time and at least 1 month ago, (2) having 24 or higher score from Standardized Mini‐Mental Test, (3) having in affected UE at least 20° of active wrist extension starting from the full flexion, 10° of active extension or abduction in the thumb, and 10° of active extension in the metacarpophalangeal and interphalangeal joints of the other fingers, (4) scoring below 2.5 points in both sections of Motor Activity Log‐28 (MAL‐28), (5) being able to stand for 2 min without assistance from a person (can get support from the UE), and (6) participants in the telerehabilitation group must have necessary technological device (telephone or personal computer) and the have knowledge to use that technological device, or are willing to learn how to use that they have technological device if they do not know. Participants were excluded from the study if: (1) they had spasticity in any joint of the UE and scored 2 or higher according to the Modified Ashworth Scale, (2) they had pain in the affected UE and recently scored average 4 or higher according to the Visual Analog Scale which is scored 0–10, (3) they participated in any ongoing rehabilitation program, and (4) having any other disease that prevents participation in the rehabilitation program.

The study was approved by the Clinical Research Ethics Committee of Gazi University and registered at ClinicalTrials.gov (ClinicalTrial.gov ID: NCT05128461) before the recruitment started. All procedures were in accordance with the Declaration of Helsinki and written informed consent was obtained from all participants.

### 2.2 Study design

The study was planned as a randomized controlled 1:1 allocation ratio evaluator‐blind study. Stroke patients were divided into two groups: the m‐CIMT based telerehabilitation group (Tele‐CIMT) and the home exercise group (control group [CG]) using the minimization method of Covariate Focused Randomization (Scott et al., 2002). Minimization is a randomization method that aims to produce the best overall balance between treatment groups according to patient characteristics (Taves, 2010). The allocation ratio was 1:1. All participants underwent a baseline and a post‐training evaluations face to face. All assessments were done by two physiotherapists who were blinded to the patient groups.

### 2.3 Intervention

#### 2.3.1 Modified‐constraint induced movement therapy‐based telerehabilitation

After the evaluations were completed, the basic goals and principles of m‐CIMT were explained to the patients in the Tele‐CIMT group. Afterward, training was given about the Zoom or Skype applications that the patients would use for videoconference. Then, three to five activities that the patient wanted to do but could not do after the stroke were selected to be used in shaping and task practice (Supporting Information). These tasks were selected from the Motor Activity Log or from ADLs identified by patient (Page et al., 2013). After that, behavioral contact (D. Morris et al., 2006; Page et al., 2013) was applied to all participants. Finally, a set of materials, including a mitt to be used to restrain their less affected UEs and materials to be used in intervention sessions, were delivered to the patients. Some materials such as spoons, forks, and knives that could be found in every home were provided by the patient.

Sirtori et al.'s (2009) meta‐analysis examining the duration of application of m‐CIMT showed that when the total application time is equal to or less than 30 h, there is a significant improvement in the motor function of the paretic UE, but no additional improvement when the application time exceeds 30 h. In another study, it was reported that more than 80% of patients, who underwent CIMT, preferred training consisting of shorter sessions but completed in more weeks, rather than long sessions and 2 week applications as in the original protocol of CIMT (Blanton & Wolf, 1999). From this point of view, Tele‐CIMT was applied to the participants for 5 weekdays for 3 weeks, 90 min for each session. The Tele‐CIMT group received treatment via videoconference. The treatment was conducted for all participants by the same physiotherapist, and the physiotherapist attended all online videoconference sessions. A caregiver of the patient was asked to be present in the room throughout the treatment, both for safety reasons and to assist the patient during the exercises (e.g., to give the patient an exercise material that he dropped on the floor or to bring materials such as a spoon to be obtained from patient's home during the exercise). When there was a technical problem, such as an internet connection problem, the patient was called by phone and guidance was given. In addition, patients were asked to wear a mitt on their less affected hand during treatment and while awake, 5 h a day, 5 weekdays. During the treatment sessions, shaping, task practice, home diary, behavioral contact, home practice, and daily schedule included in the CIMT protocol were applied to the patients (D. Morris et al., 2006; Page et al., 2013).

#### 2.3.2 Home exercise program

Participants in both groups were asked to perform a home exercise program consisting of 10 basic exercises aimed at improving UE range of motion, active movement, balance, and walking for 3 weeks, 5 weekdays (Supporting Information). All patients were shown the exercises and given a brochure with photos and explanations on how to do them. Also, the patients were asked to mark whether they did their exercises on the diary in the brochure. They were asked to bring this brochure on the day of the posttreatment evaluation, and it was checked whether they did the exercises.

### 2.4 Outcome measures

The primary outcome measure was UE motor functions measured with the Stroke Rehabilitation Assessment of Movement Scale (STREAM), Fugl‐Meyer Upper Extremity Motor Evaluation Scale (FM‐UE), Wolf Motor Function Test (WMFT), and 9‐Hole Peg Test (9‐HPT). Secondary outcome measures included grip strengths, pinch strengths, Motor Activity Log‐28 (MAL‐28), and Functional Independence Measure (FIM).

The modified Rankin score was used to determine the patient's disability level. In the scale, disability level is evaluated at seven levels, and ambulation is evaluated at six levels. Completely normal functional status is defined by a score of 0 and death by a score of 6 (Banks & Marotta, 2007).

The STREAM was used to evaluate the functional movement ability and mobility levels of the patients. STREAM consists of 10 tests that evaluate voluntary movement and basic mobility activities in the upper and lower extremities. Scoring is done by taking the quantity and quality of the action as criteria. The highest score that can be obtained from the scale is 70, and higher scores indicate less motor impairment (Daley et al., 1999).

The FM‐UE was used to evaluate the UE motor functions of the patients. FM‐UE, consisting of 33 items, evaluates the movement, coordination, and reflexes of the shoulders, elbows, forearms, wrists, and fingers. The highest score of 66 can be obtained from this scale, and higher scores indicate better motor functions (Fugl‐Meyer et al., 1975).

The WMFT was used to assess motor ability in patients. In our study, the modified WMFT of the scale was used. In the test, which consists of 17 different activities, two items evaluate muscle strength. For the other 15 functional activities, data are collected in two areas, functional ability and performance time. The functional ability score is calculated by scoring the 15 functional activities evaluated between 0–5 points and taking the average of the total score. Higher scores indicate better functional ability. In the performance time section, the time taken for each activity is recorded (Morris et al., 2001).

The 9‐HPT, which is valid and reliable in stroke, was used to assess fine dexterity. In our study, the test was applied twice, and the best time was recorded as second (Chen et al., 2009).

The patients’ gross hand grip strengths were evaluated using J‐Tech and pinch grip strengths (bipod, tripod, and lateral) were evaluated using the Baseline Hydraulic Pinch Meter. All measurements were performed while the patient was seated in the upright position and the patient's shoulder was adduction, the forearm was in the neutral position, the elbow was 90° flexion, and the wrist was 0–30° extension and 15° ulnar deviation as recommended by the American Association of Hand Therapists (Fess, 1992).

The MAL‐28 was used to evaluate how often and how well the patient used the affected extremity in ADLs. The scale consists of two parts. In the first part, it is evaluated how often the patient uses the affected UE in 28 daily living activities (amount of use [AOU]). In the second part, it is evaluated if patient does, how successful patient is in doing the activity (quality of use [QOU]). Scoring is done by the patients themselves. The average of the total scores in both sections was used (Hseyinsinoğlu & Krespi, 2011).

The FIM was used to determine the level of independence of patients in basic ADLs. The scale consists of six sub‐headings and a total of 18 items. Scores that can be obtained from the scale range from 18–126, and higher scores indicate that the individual is more independent in daily life (Hall et al., 1993; Küçükdeveci et al., 2001).

### 2.5 Statistics

The sample size was calculated based on the significant improvement of the WMFT functional ability test score observed in a similar intervention study (Hsieh et al., 2014). Their findings provided a Cohen's *d* effect size of 0.468. To achieve 80% power with a two‐sided level of 5%, the total sample size was estimated at a minimum of 16 (n:8 per group) for both effect sizes using G*Power 3.1 power analysis software (Faul et al., 2007). Assuming a 15% dropout rate, we recruited 10 participants per group.

IBM Statistics SPSS v21.0 (IBM Corp.) was used for statistical analysis. A Shapiro–Wilk test was used to determine the normality of the data. Because data do not match the normal distribution, logarithmic transformation was applied. After that, the 2 × 2 factorial analysis of variance was used with time (pre vs. post) and group (Tele‐CIMT group vs. CG) as the independent variables for the outcome variables. Post hoc comparisons were assessed using Bonferroni corrections. For non‐normally distributed outcome, variables were presented as median and interquartile range (IQR). In all statistical analyses, the significance level was set at *p* < .05.

## 3 RESULTS

**FIGURE 1:**
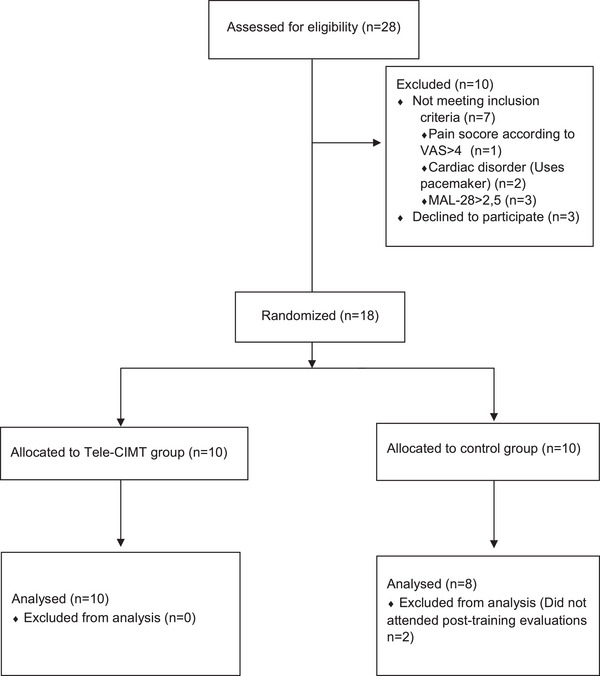
Participants flow through the study. Consolidated Standards of Reporting Trials (CONSORT) flow chart. VAS, Visual Analog Scale; MAL, motor activity log; Tele‐CIMT, modified‐constraint induced movement therapy‐based telerehabilitation.

The flow chart of the study is shown in Figure 1. Twenty‐eight stroke patients were assessed for eligibility, and 18 stroke patients were included. The demographic variables, clinical characteristics, and baseline characteristics are presented in Tables 1 and 2. (*p* > .05).

**TABLE 1:** Comparison of demographic and clinical characteristics of the participants.

	Tele‐CIMT (*n* = 10) Median (IQR)	CG (*n* = 8) Median (IQR)	*p*
**Age (years)**	51.50 (39.50–61.75)	51.50 (42.00–63.25)	.894
**BMI (kg/m^2^)**	25.73 (23.58–27.65)	25.72 (22.88–29.25)	1.000
**Sex, F/M (%male)**	2/8 (%80)	2/6 (%75)	1.000
**Affected side, right/left (%right)**	5/5 (%50)	5/3 (%62,5)	1.000
**Disease duration (month)**	10.50 (2.75–15.37)	10.00 (3.37–15.5)	.893
**Stroke type, ischemic /hemorrhagic (%ischemic)**	7/3 (%70)	6/2 (%75)	1.000
**mRS**			
**2**	6 (%60)	6 (%75)	.638
**3**	4 (%40)	2 (%25)	

*Note*: Data are presented as median (IQR) for non‐normally distributed: *p *< .05.

Abbreviations: BMI, body mass index; CG, control group; F, female; IQR, interquartile range; M, male; mRS, modified ranking score; Tele‐CIMT, modified‐constraint induced movement therapy‐based telerehabilitation.

**TABLE 2:** Comparison of base characteristics of the participants.

	Tele‐CIMT (*n* = 10) Median (IQR)	CG (*n* = 8) Median (IQR)	*p*
**STREAM**			
Upper extremity	15.00 (11.00–16.25)	14.50 (14.00–16.00)	.652
Lower extremity	17.50 (16.75–19.00)	17.00 (17.00–18.75)	.817
Basic Mobility	28.50 (24.75–30.00)	29.50 (26.50–30.00)	.545
Total score	62.00 (55.25–64.0)	61.50 (59.25–63.00)	.489
**FM‐UE**	54.50 (49.75–57.25)	55.00 (53.00–56.75)	.721
**WMFT**			
Functional ability	3.96 (3.43–4.22)	4.08 (3.36–4.25)	.722
Performance time	3.24 (1.93–5.15)	3.48 (2.04–4.66)	.824
**9‐HPT**	67.03 (33.93–208.25)	47.42 (36.87–157.00)	.894
**Gross grip strength (N)**	236.50 (128.7–328.00)	244.00 (152.80–355.50)	.929
**Bipod pinch (N)**	8.00 (4.75–12.75)	8.50 (5.00–14.00)	.822
**Tripod pinch (N)**	10.50 (5.75–14.25)	12.50 (5.25–14.00)	.823
**Lateral pinch (N)**	15.50 (8.00–22.00)	18.00 (9.00–22.00)	.751
**MAL‐28**			
AOU	1.31 (0.96–2.18)	1.30 (1.13–1.91)	.894
QOU	1.38 (0.92–2.31)	1.33 (1.03–2.12)	1.000
**FIM‐**	114.50 (111.75–115.00)	113.5 (107.25–114.00)	.340

*Note*: Data are presented as median (IQR) for non‐normally distributed: *p *< .05.

Abbreviations: 9‐HPT, 9‐hole peg test; AOU, amount of use; CG, control group; FIM, functional independence scale; FM‐UE, Fugl‐Meyer upper extremity motor scale; IQR, interquartile range; MAL‐28, motor activity log‐28; QOU, quality of use; STREAM, stroke rehabilitation assessment of movement scale; Tele‐CIMT, modified‐constraint induced movement therapy‐based telerehabilitation; WMFT, Wolf motor function test.

Throughout the intervention, participants in the Tele‐CIMT group fully attended regularly in all telerehabilitation sessions. It was determined that the patients in the CG did their home exercises completely 5 days a week for 3 weeks. During the telerehabilitation sessions, there were no technical problems and so on that would prevent the session from being performed.

At the end of the 3 weeks, there were significant interaction effects (time × group) for STREAM‐upper extremity, STREAM‐lower extremity, STREAM‐total score FM‐UE, WMFT‐Functional ability, WMFT‐performance time, 9‐HPT, gross grip strength, bipod pinch strength, tripod pinch strength, and lateral pinch strength, MAL‐28‐AOU, MAL‐28‐QOU, and FIM (*F* = 18.222, *p* = .001, *F* = 11.305, *p* = .004, *F* = 13.611, *p* = .002, *F* = 19.530, *p* = .001, *F* = 50.671, *p* = .001, *F* = 29.341, *p* = .001, *F* = 6.874, *p* = .019, *F* = 5.507, *p* = .032, *F* = 25.995, *p* = .001, *F* = 7.013, *p* = .018, *F* = 7.766, *p* = .013, *F* = 21.124, *p* = .001, *F* = 13.857, *p* = .002, and *F* = 9.898, *p* = .006, respectively) (Table 3). Post hoc analyses revealed that the Tele‐CIMT group significantly improved STREAM‐Upper extremity, STREAM‐Lower extremity, STREAM‐Total score FM‐UE, WMFT‐Functional ability, WMFT‐Performance time, gross grip strength, bipod pinch strength, tripod pinch strength, and lateral pinch strength, MAL‐28‐AOU, MAL‐28‐QOU, and FIM (p < 0.05). (Table 3).

**TABLE 3:** Treatment effects on upper extremity functions, grip strengths, and activities of daily living for the Tele‐CIMT (modified‐constraint induced movement therapy‐based telerehabilitation) group and control group (CG) at baseline and 3 week follow‐up.

		Pretest Median (IQR)	Posttest Median (IQR)	*p* (within group)	*F*	*p*	*ηp* ^2^
**STREAM**							
Upper extremity	Tele‐CIMT	15.00 (11.00–16.25)	19.50 (18.00–20.00)	.001*	18.222	.001*	0.532
	CG	14.50 (14.00–16.00)	15.00 (14.25–16.75)	.626			
Lower extremity	Tele‐CIMT	17.50 (16.75–19.00)	20.00 (19.00–20.00)	.001*	11.305	.004*	0.414
	CG	17.00 (17.00–18.75)	17.50 (17.00–19.00)	.197			
Basic mobility	Tele‐CIMT	28.50 (24.75–30.00)	29.50 (28.75–30.00)	.068	1.703	.210	0.096
	CG	29.50 (26.50–30.00)	29.50 (26.5–30.00)	1.000			
Total score	Tele‐CIMT	62.00 (55.25–64.0)	68.50 (66.25–69.25)	.001*	13.611	.002*	0.460
	CG	61.50 (59.25–63.00)	62.00 (60.25–63.75)	.564			
**FM‐UE**	Tele‐CIMT	54.50 (49.75–57.25)	64.00 (62.75–65.00)	.001*	19.530	.001*	0.550
	CG	55.00 (53.00–56.75)	55.50 (54.25–57.00)	.704			
**WMFT**							
Functional ability	Tele‐CIMT	3.96 (3.43–4.22)	4.93 (4.65–4.95)	.001*	50.671	.001*	0.760
	CG	4.08 (3.36–4.25)	4.13 (3.38–4.31)	.700			
Performance time	Tele‐CIMT	3.24 (1.93–5.15)	1.42 (1.20–1.85)	.001*	29.341	.001*	0.647
	CG	3.48 (2.04–4.66)	3.35 (2.02–4.41)	.737			
**9‐HPT**	Tele‐CIMT	67.03 (33.93–208.25)	36.70 (20.63–56.75)	.001*	6.874	.019*	0.301
	CG	47.42 (36.87–157.00)	46.80 (35.94–111.11)	.557			
**Gross grip strength (N)**	Tele‐CIMT	236.50 (128.7–328.00)	268.00 (184.00–362.75)	.001*	5.507	.032*	0.256
	CG	244.00 (152.80–355.50)	244.30 (176.07–364.00)	.529			
**Bipod pinch (N)**	Tele‐CIMT	8.00 (4.75–12.75)	12.50 (9.00–18.00)	.001*	25.995	.001*	0.619
	CG	8.50 (5.00–14.00)	8.50 (5.75–14.00)	.682			
**Tripod pinch (N)**	Tele‐CIMT	10.50 (5.75–14.25)	15.50 (10.00–18.25)	.001*	7.013	.018*	0.305
	CG	12.50 (5.25–14.00)	12.50 (6.00–14.75)	.602			
**Lateral pinch (N)**	Tele‐CIMT	15.50 (8.00–22.00)	19.00 (10.75–25.00)	.001*	7.766	.013*	0.327
	CG	18.00 (9.00–22.00)	18.00 (9.25–22.00)	.817			
**MAL‐28**							
AOU	Tele‐CIMT	1.31 (0.96–2.18)	3.73 (3.45–4.78)	.001*	21.124	.001*	0.569
	CG	1.30 (1.13–1.91)	1.43 (1.26–2.32)	.218			
QOU	Tele‐CIMT	1.38 (0.92–2.31)	3.97 (3.38–4.61)	.001*	13.857	.002*	0.464
	CG	1.33 (1.03–2.12)	1.58 (1.28–2.38)	.274			
**FIM**	Tele‐CIMT	114.50 (111.75–115.00)	123.00 (121.50–124.25)	.001*	9.898	.006*	0.382
	CG	113.5 (107.25–114.00)	114 (108.75–115.00)	.798			

*Note*: Data are presented as median (IQR) for non‐normally distributed: *p *< .05.

Abbreviations: 9‐HPT, 9‐hole peg test; AOU, amount of use; FIM, functional independence scale; FM‐UE, Fugl‐Meyer upper extremity motor scale; IQR, interquartile range; MAL‐28, motor activity log‐28; QOU, quality of use; STREAM, stroke rehabilitation assessment of movement scale; WMFT, Wolf motor function test.

## 4 DISCUSSION

Learned non‐use is an important factor that complicates the recovery of motor functions in the UE in stroke patients. CIMT is one of the high‐evidence methods used in UE rehabilitation after stroke, based on forcing the affected UE to use it (Sirtori et al., 2009). To the best of our knowledge, this is the first randomized controlled study to investigate the effects of m‐CIMT based telerehabilitation applied via videoconference on UE functions and ADLs in stroke patients. According to the results of our study, it was determined that the UE motor functions, fine dexterity, and grip strength improved, the amount and quality of use of the UE in daily living activities of the patients increased, and thus the functional independence level of the individual in activities of daily life significantly improved with Tele‐CIMT.

There was an improvement in the voluntary movements of the lower extremity, which we evaluated with STREAM, in the Tele‐CIMT group, but these improvements were not observed in the CG. Information obtained from studies in the literature shows that there is a positive relationship between UE functions and lower extremity functions (Arslan et al., 2021; Fujita et al., 2015). Therefore, we think that the improvement in the Tele‐CIMT group may be related to the improvements in the UE functions.

Pickett et al. (2007) applied CIMT via videoconference to two patients who had previously applied CIMT in a clinical setting. At the end of the study, the researchers reported that similar improvements were obtained in the UE motor functions and gross manual dexterity of the patients using both methods. In addition, they showed similar improvements in ADLs, grip strength, and quality of life. Similar results were also obtained in our study. Additionally, in this study, the pinch grip strength required for fine grips was also evaluated, and the improvement of these have been shown in the Tele‐CIMT group in our study.

In the study by Uswatte et al. (2021), they compared the results of CIMT and CIMT‐based telerehabilitation applications, which they called Tele‐AutoCITE. The Tele‐AutoCITE is an automated computerized system with 11 tasks similar to the CIMT activities they practice in the clinical setting. While this system gives visual feedback to the patient about the patient's performance, it also allows the therapist to intervene in the exercises during the training by connecting via videoconference. It was reported that the AOU of the affected extremity in ADLs increased in both groups and that the application of CIMT based telerehabilitation would provide similar developments. When we compare these results with the results of our study, it is seen that the AOU of the affected extremity in ADLs increased in our study as well. Furthermore, in our study, it is seen that more shaping and task activities are used, the difficulty of shaping and task activities is systematically increased, and there are more comprehensive outcome measures compared to their study.

The other study, which was conducted by Page and Levine (2007), examined the effectiveness of m‐CIMT based telerehabilitation applied via videoconference and stated that the AOU of the affected extremities of the patients in daily life and their motor functions improved similarly to our results. In our study, m‐CIMT was also used, but Page et al. applied a treatment program of 30 min a day, 3 days a week, for 10 weeks, while in our study, a more intense treatment program of 90 min a day, 5 days a week, for 3 weeks was applied. Another difference in our study is that fine dexterity, gross grip strength, and pinch grip strength were evaluated.

Smith and Tomita (2020), who examined the effects of m‐CIMT based telerehabilitation as in our study, evaluated UE motor functions and ADLs in stroke patients. They stated improvement in these parameters, like our study. However, they applied a combination of m‐CIMT and m‐CIMT based telerehabilitation. In our study, we only applied m‐CIMT with telerehabilitation. Similar to the other studies, they did not measure the fine dexterity, gross grip strength, and pinch grip strength.

As mentioned above, there are a few studies examining the effects of CIMT and m‐CIMT based telerehabilitation in stroke patients. When these studies are examined, it is seen that the effects of CIMT or m‐CIMT‐based telerehabilitation on UE motor functions (Page & Levine, 2007; Pickett et al., 2007; Smith & Tomita, 2020), gross manual dexterity (Pickett et al., 2007), gross grip strength (Pickett et al., 2007), and ADLs (Smith & Tomita, 2020) were evaluated, and similar developments were observed with our study. One of these studies was a randomized controlled proof of concept trial, and it investigated only the AOU of the affected UE. However, UE motor functions and ADLs were not evaluated (Uswatte et al., 2021). Thus, our study is the first randomized controlled study to investigate the effects of m‐CIMT based telerehabilitation applied via videoconference on UE functions and ADLs in stroke patients. If we examine the sample sizes in these studies, two of them were case studies, and interventions were applied to two patients in one (Pickett et al., 2007) and four patients in the other (Page & Levine, 2007). From this point of view, it is clearly seen that our study differs from other studies because it is a randomized controlled study, comprehensive evaluations were made for UE motor functions and ADLs, pinch grip strength and fine dexterity were evaluated, which were not evaluated in other studies, and even lower extremity movements and mobility were evaluated.

The minimal clinically important difference (MCID) of the STREAM were 2.2, 1.9, and 4.8 points for the UE subscale, lower‐extremity subscale, and mobility subscale, respectively (Hsieh et al., 2008). The 4.8, 1.7, and 3.1 points increase was found in the STREAM of the Tele‐CIMT group. The MCID of the FM‐UE was stated as 4.25–7.25 points (Page et al., 2012). In our study, 10.6 points increase was found in the FM‐UE of the Tele‐CIMT group. The MCID of WMFT‐performance time was stated as 1.5–2 s and WMFT‐functional ability score was stated as 0.2–0.4 points (Lin et al., 2009). According to the findings of our study, 2.28 s decrease was found in the WMFT‐performance and 1 point increase was found in the WMFT‐functional ability score of the Tele‐CIMT group. The MCID of the 9‐HPT was stated as 32.8 s (Chen et al., 2009). In our study, 71.64 s decrease was found in the 9‐HPT of the Tele‐CIMT group. All of these results indicated that the UE function in the Tele‐CIMT group was improved clinically. The MCID of FIM was stated as 22 points. According to our results, there is 12.9 points increase in the FIM of the tele‐CIMT group. Although the increase in FIM is statistically significant, it is not clinically meaningful. The reason behind this may be that FIM does not only include ADLs related to the UE, but our intervention is not only aimed at the UE. This situation may be taken into consideration in future studies.

### 4.1 Limitations

There were some limitations to this study. First, since the study was conducted during the COVID‐19 pandemic, m‐CIMT was administered only through telerehabilitation. Therefore, future studies are needed to compare the results of m‐CIMT applied face‐to‐face in the clinical setting and m‐CIMT applied through telerehabilitation. In our study, there was no long‐term follow‐up. Therefore, future studies are needed to assess long‐term follow‐up. Another limitation of our study was that we did not evaluate the patient's satisfaction levels. We recommend that the satisfaction level be questioned in future studies. Finally, although seemingly adequately powered, the sample size in this study may still limit the generalizability of the study results. Future studies can be planned more comprehensively, considering the limitations of this study.

## 5 CONCLUSION

Results of this study showed that the Tele‐CIMT program may have beneficial effects on UE motor functions, grip strength, fine dexterity, the amount and quality of use of the UE in daily living activities, and daily living activities in stroke patients. Moreover, no adverse events or injuries were reported by the patients during the telerehabilitation sessions in our study. These findings indicate that the Tele‐CIMT protocol allows successful and safe training of UE motor function through telerehabilitation. Therefore, this training program will guide clinicians interested in UE training for stroke patients. However, although the results of our study show that the Tele‐CIMT protocol was successful, further studies with larger participants and comparison with face‐to‐face treatment options are needed for more definitive evidence, as stated in the limitations.

## AUTHOR CONTRIBUTIONS

**Fettah Saygili**: Conceptualization; investigation; writing—original draft; methodology; validation; visualization; writing—review and editing; formal analysis; project administration; data curation; supervision; resources. **Arzu Guclu‐Gunduz**: Conceptualization; investigation; writing—original draft; methodology; writing—review and editing; project administration. **Sefa Eldemir**: Investigation; writing—original draft; Formal analysis. **Kader Eldemir**: Investigation; writing—original draft. **Cagla Ozkul**: Conceptualization; methodology. **Gorkem Tutal Gursoy**: Supervision; resources.

## FUNDING INFORMATION

The author(s) received no financial support for the research, authorship, and/or publication of this article.

## CONFLICT OF INTEREST STATEMENT

The authors declared that there are no conflicts of interest.

### PEER REVIEW

The peer review history for this article is available at https://publons.com/publon/10.1002/brb3.3569.

## Supporting information

Supporting Information

## Data Availability

Data sharing not applicable to this article as no datasets were generated or analyzed during the current study.
