# Supplementary material for: Effects of modified‐constraint induced movement therapy based telerehabilitation on upper extremity motor functions in stroke patients
Source: Brain Behav. 2024 Jun 14;14(6):e3569. doi: 10.1002/brb3.3569 (PMC11177030; doi:10.1002/brb3.3569)
Supplement: Supplementary file 1 — Supporting Information [file BRB3-14-e3569-s001.docx]

**Examples of Shaping Practice**

| 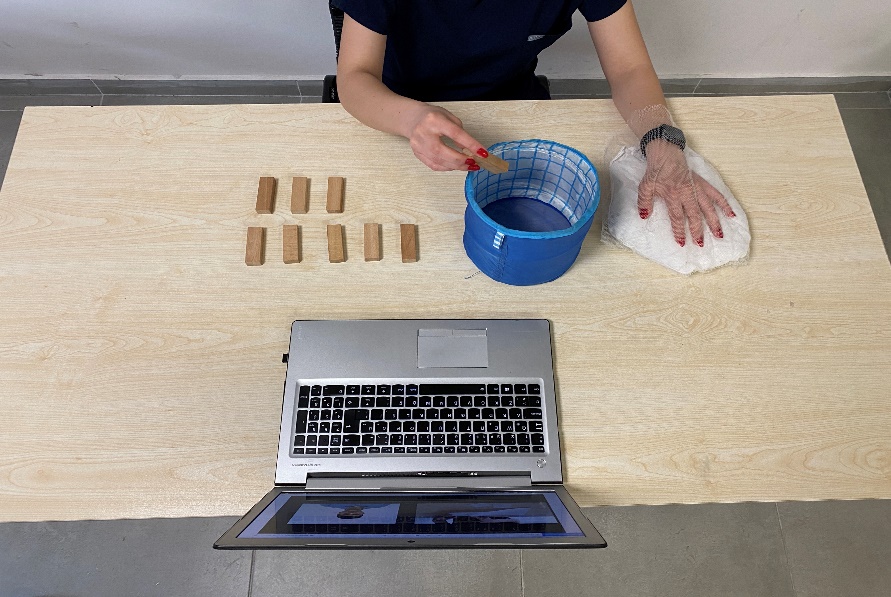 | Put the blocks into the basket  Activity: The patient was asked to grasp the wooden blocks on the table and throw them into the basket.  Complicating:  • The size of the blocks has been reduced  • The weight of the blocks has been increased  • The number of blocks to be added to the basket in the time given to completing the activity in the previous attempt has been increased.  • The time required to put the same number of blocks has been reduced   - • The basket position was positioned higher or farther forward from the patient than the intended upper extremity movement. For example, the box was placed higher to complicate upper extremity flexion. |
| --- | --- |
| 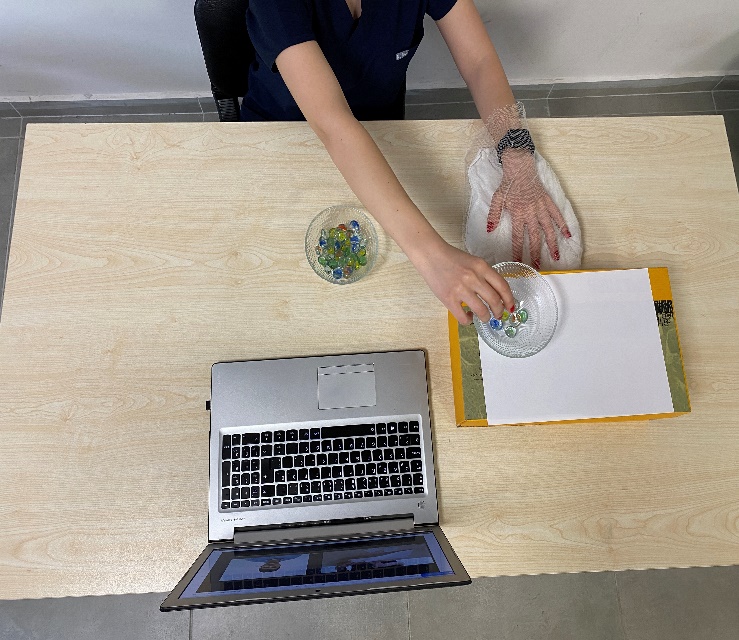 | Carrying marbles  Activity: The patient was asked to put the marbles from one bowl to the other. While doing the activity, grasping was tried with all fingers in the form of holding and releasing with the thumb-index, head-middle, head-ring and thumb-little finger.  Complicating:  • Marbles have been shrunk.  • The number of marbles placed in the bowl was increased in the time given to the patient to complete the activity in the previous trial.  • The time required to put the same number of marbles in the bowl has been reduced.  The position of the bowl was changed by placing the bowl higher and farther, as seen in the picture on the right. |
| 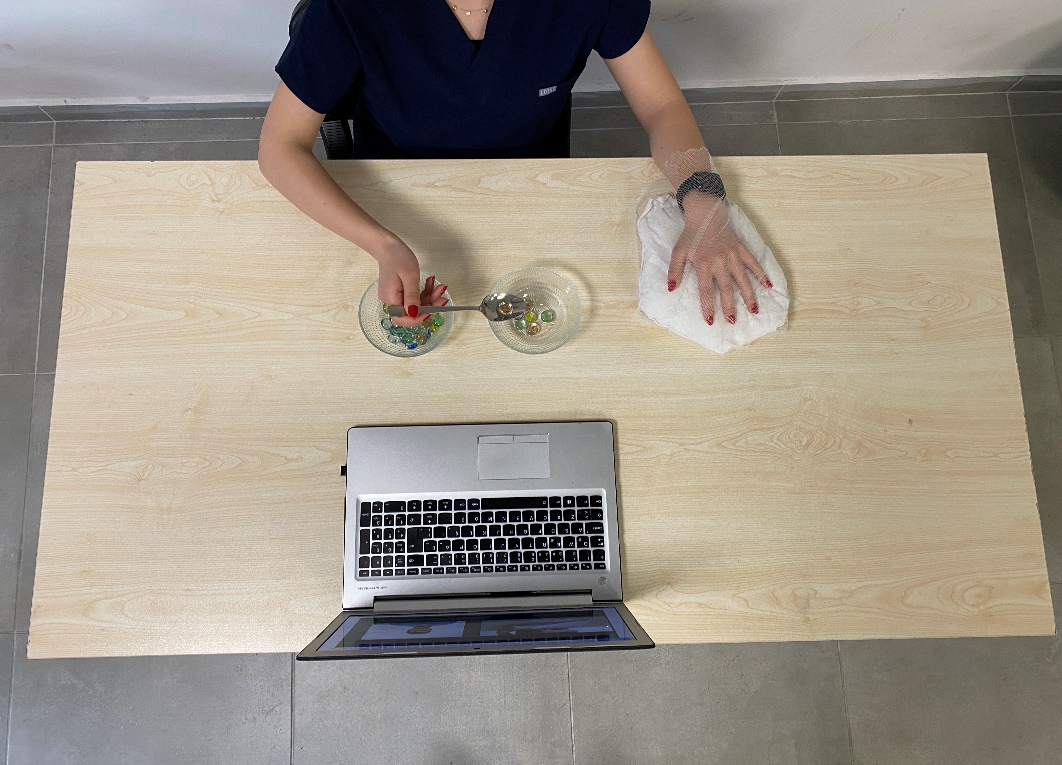 | Transfer Marbles with a Spoon  Activity: The patient was asked to take the marbles with a spoon and transfer them from one bowl to the other.  Complicating:  • Marbles have been shrunk  • The number of marbles placed in the bowl in the time given to the patient to complete the activity in the previous trial has been increased.  • Reduced the time required to put the same number of marbles in the bowl  • The bowl is positioned higher or further (right or left) from the midline. |
| 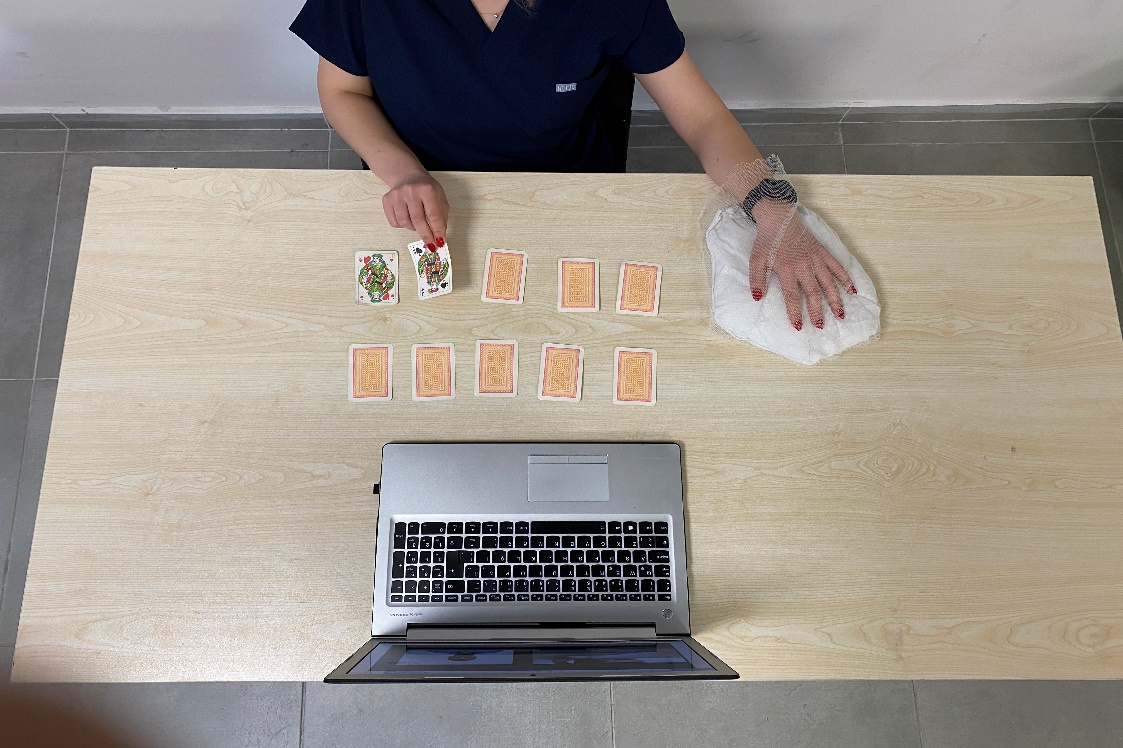 | Flipping cards  Activity: The patient is asked to turn over the cards lying on the table  Complicating:  • Cards have been shrunk  • The number of cards turned over in the time given to the patient to complete the activity in the previous trial has been increased.  • The time required to turn the same number of cards has been reduced  • The position of the cards has been changed |

**Examples of Task Practice**

| 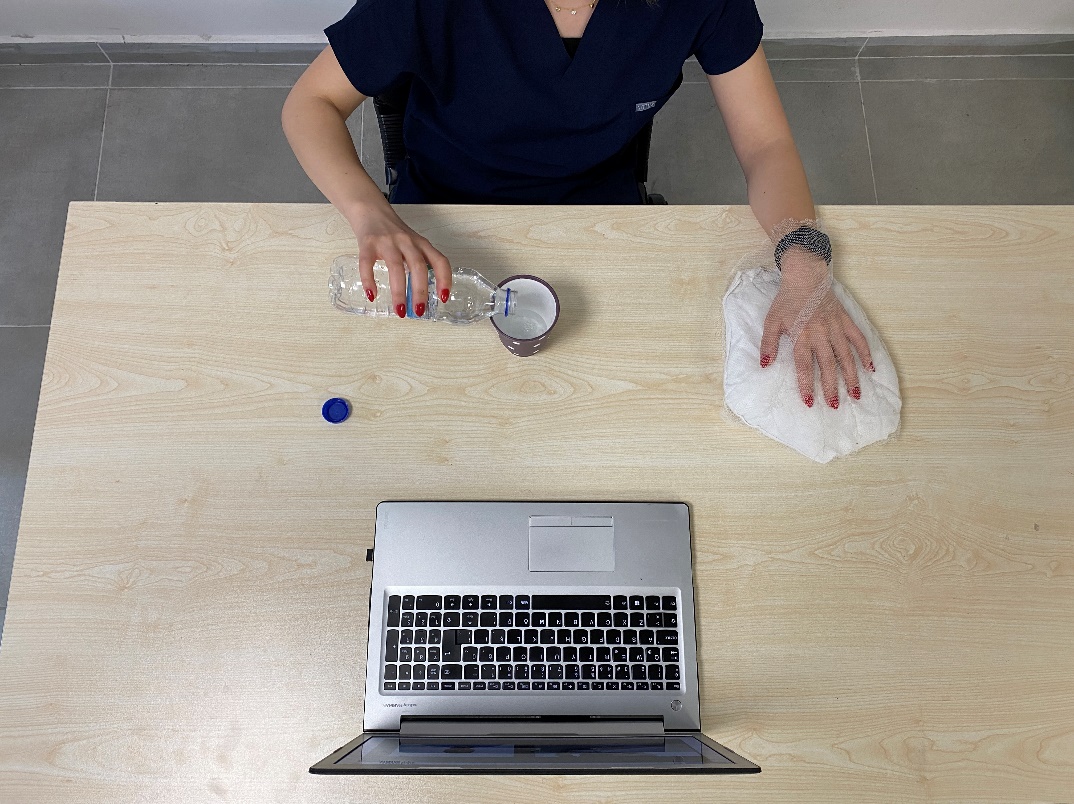 | Filling the glass with water  Activity: The patient was asked to fill a glass with water on the table.  Complicating:  • Water was filled from a heavier jug instead of a plastic bottle  • Patient filled the water while standing  • The amount of water in the bottle/jug that will be emptied in the same time has been increased  • Time taken to fill all the water in the bottle/jug into the glass has been reduced |
| --- | --- |
| 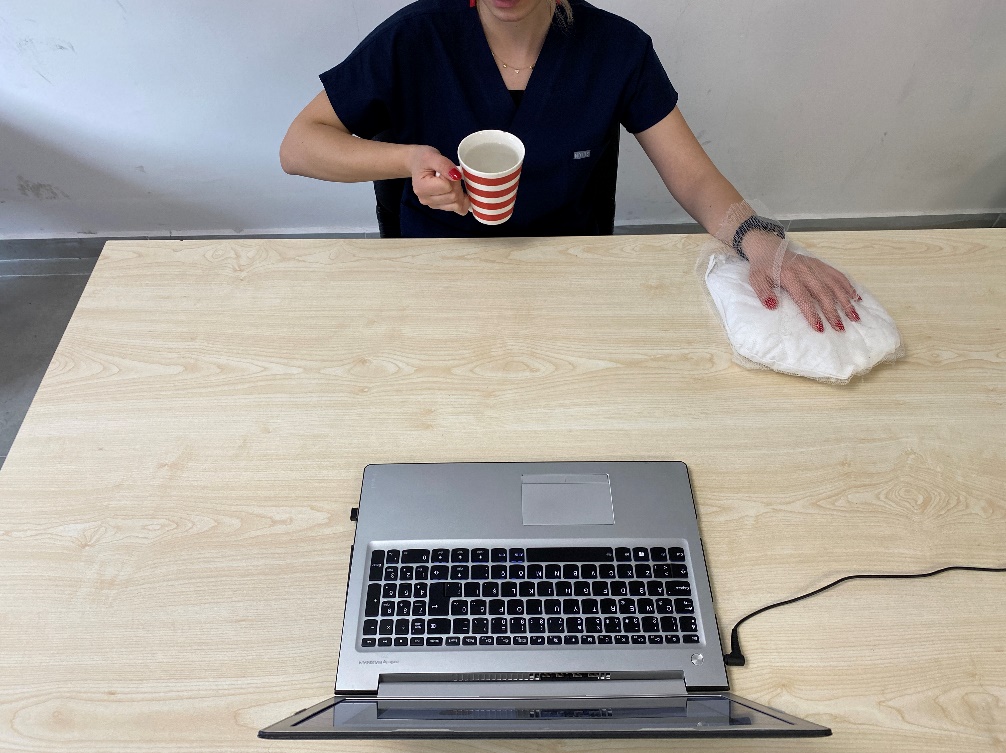 | Drinking water  Activity: The patient was asked to bring the glass of water to patients’ mouth on the table  Complicating:  • The amount of water in the glass has been increased  • Drinking water from a heavier glass  • Water was drunk while the patient was standing.  • The time required to perform the same number of moves has been reduced  Increased the amount of moves to be made in the same time |
| 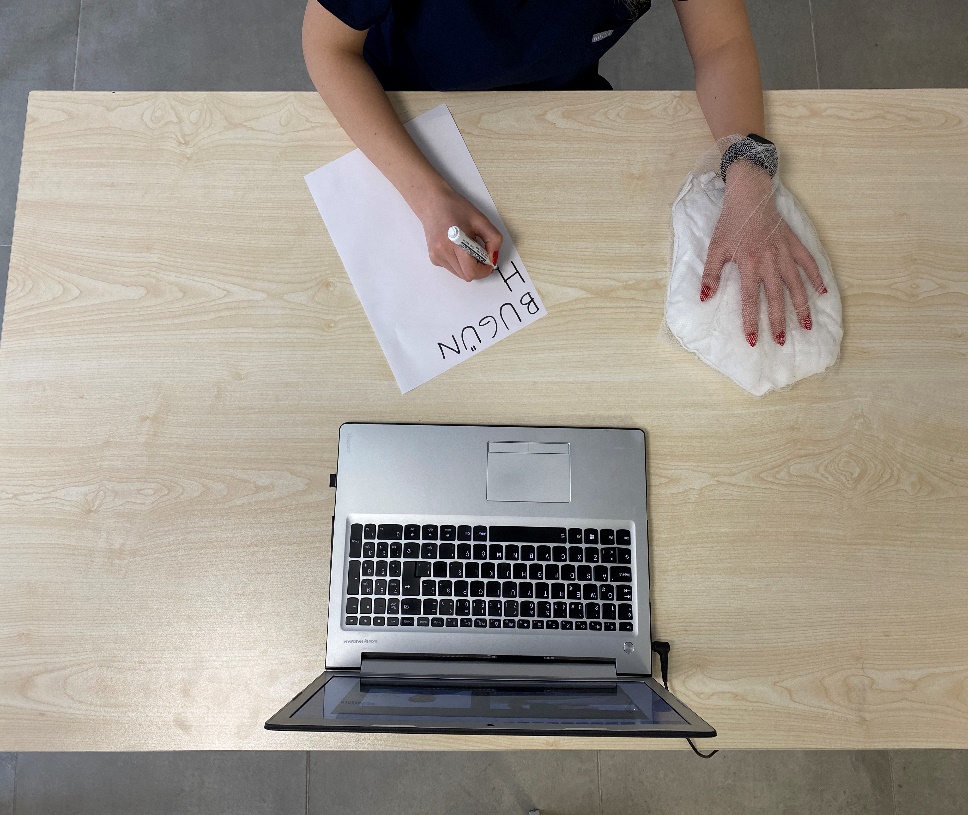 | Writing practice  Activity: The patient was asked to write down what was said on a piece of paper lying on the table.  Complicating:  • A thinner pen was used  • Written in lowercase letters  • The number of words written in the same time has been increased  • The time required to type the same number of words has been reduced  • Paper position has been changed |
| 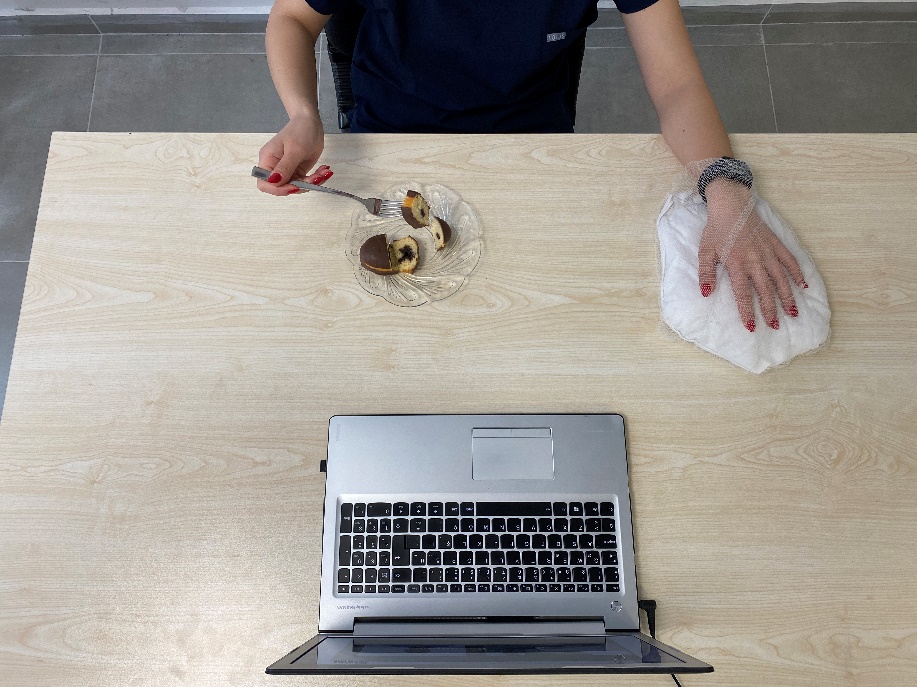 | Eating  Activity: The patient was asked to eat food with a fork or spoon  Complicating:  • Increased the weight of the Fork/Spoon  • Reduced fork/spoon handle thickness  • Advanced from solid foods to liquid foods, from large grain foods to small grain foods  • The number of food eaten in the same time has been increased  • The time required to eat the same number of foods has been reduced  • The patient ate while standing |

**Home Exercise Program**

| 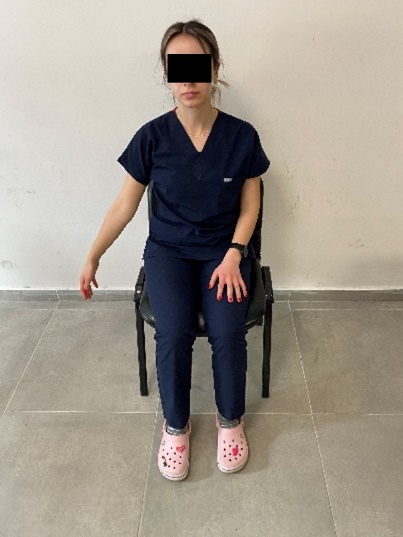 | **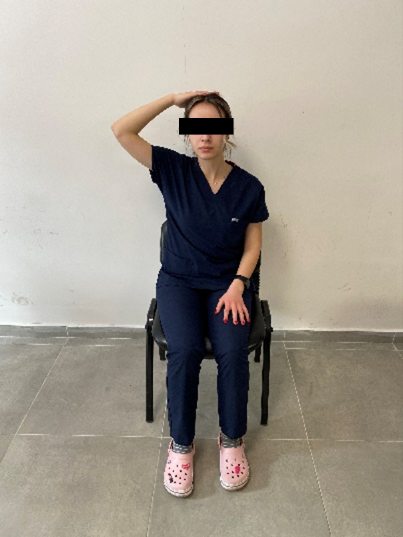** |
| --- | --- |
| Upper extremity activity, head touching | |
| 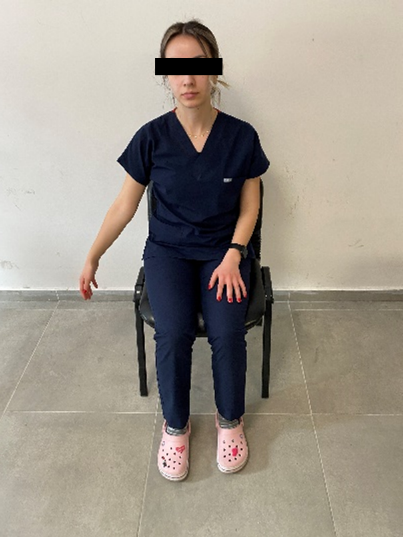 | 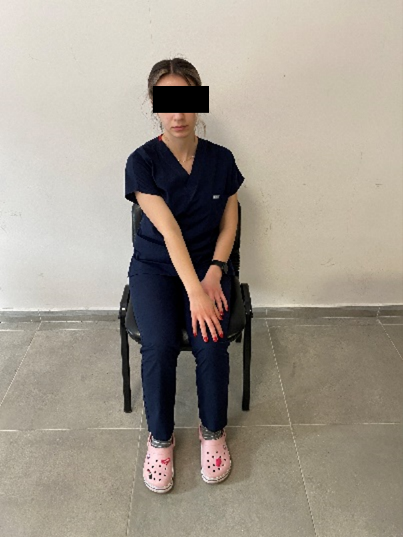 |
| Upper extremity activity, touching opposite knee | |

| 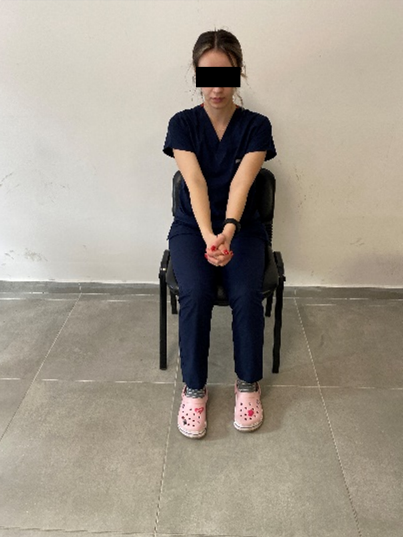 | 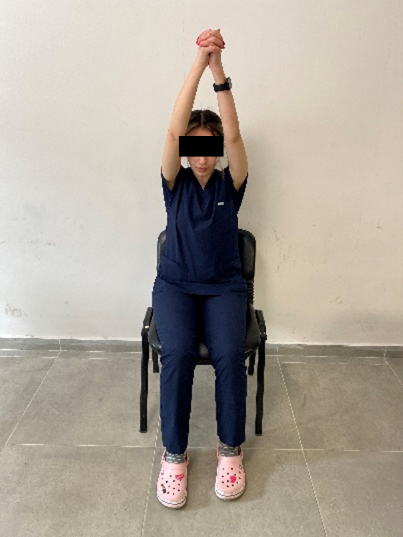 |
| --- | --- |
| Bilateral shoulder flexion-extension | |

| 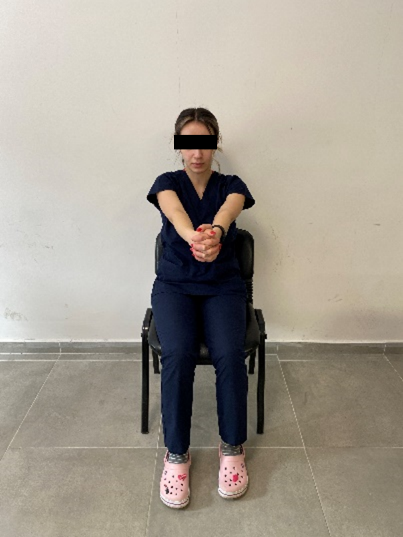 | 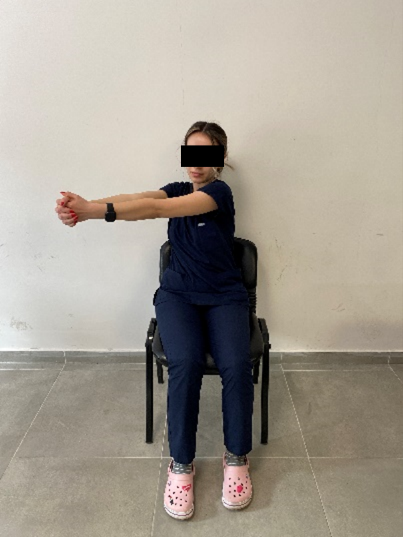 |
| --- | --- |
| Bilateral shoulder right-left rotation | |
| 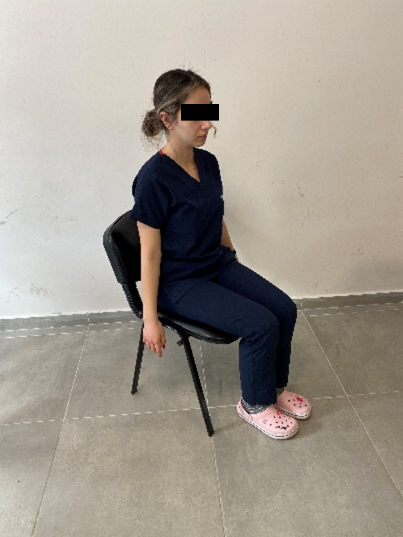 | 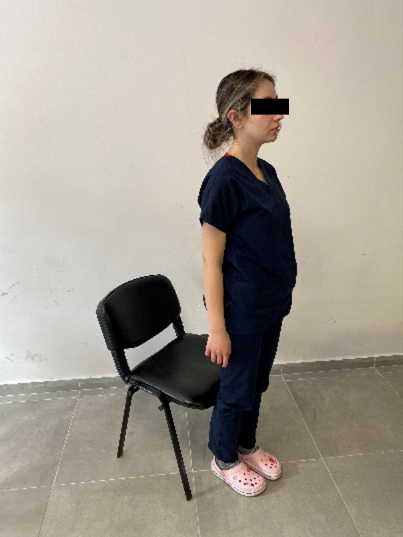 |
| Sit-Stand | |
| 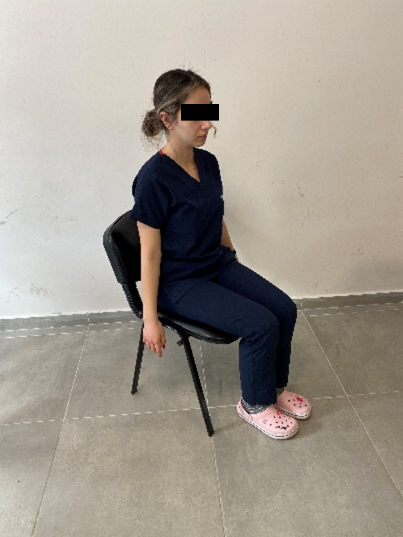 | 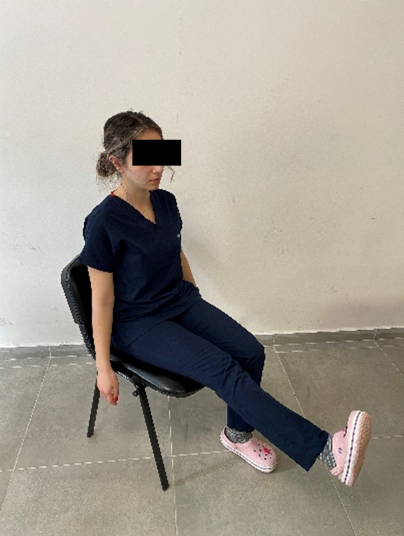 |
| Knee extension and ankle dorsiflexion training in sitting | |

| 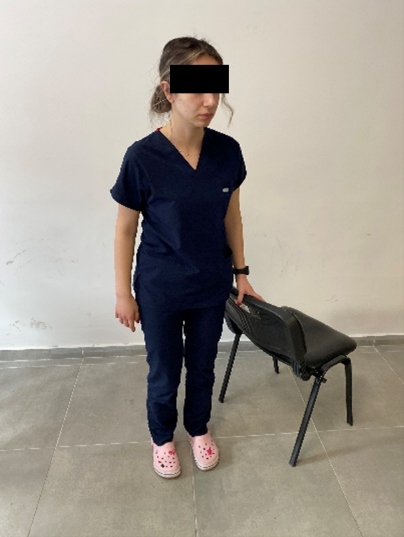 | 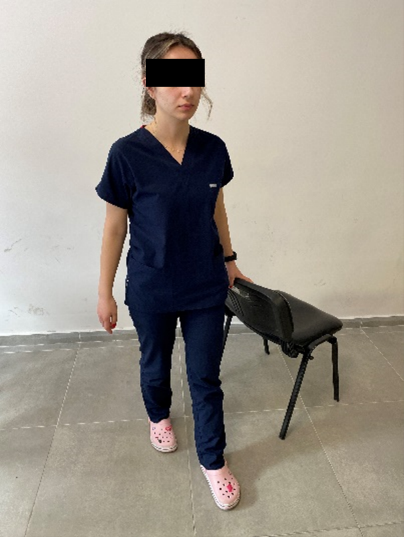 |
| --- | --- |
| Stance phase exercises, forward-backward stepping and weight transfer | |
| 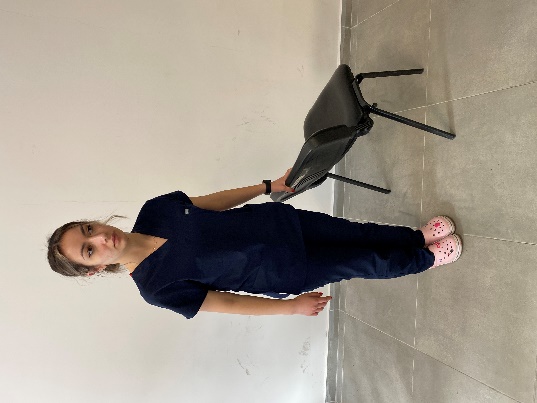 | 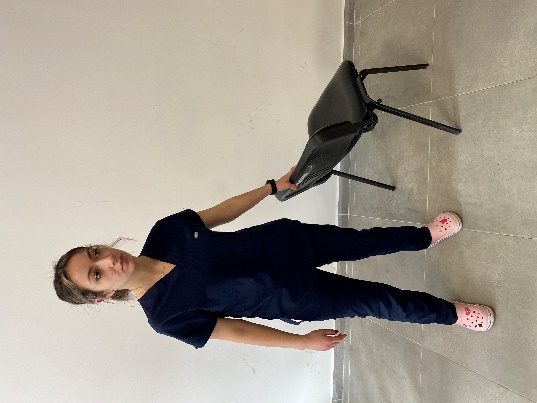 |
| Stance phase exercises, side stepping and weight transfer | |
| 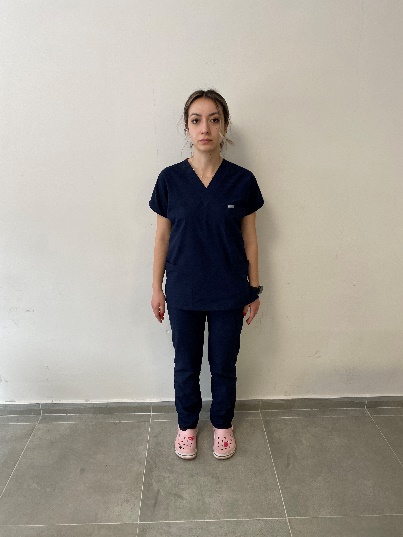 | 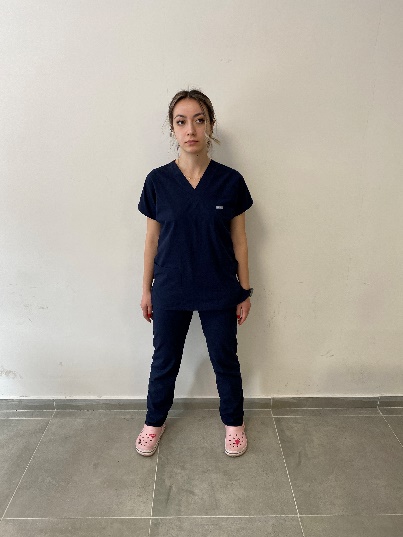 |
| Sideway walking | |
| 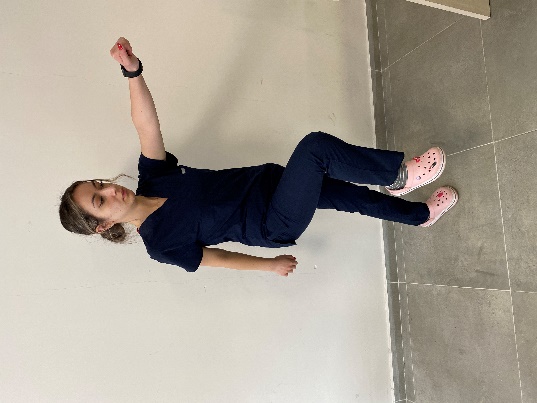 | 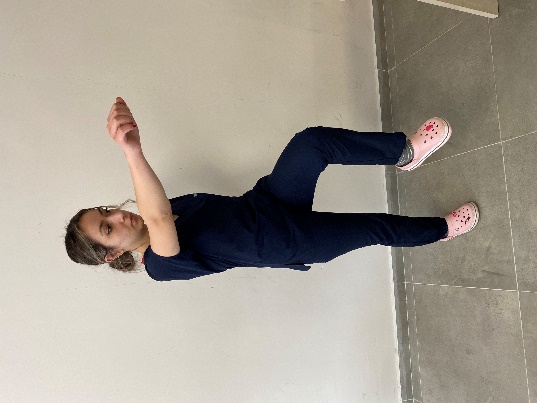 |
| Walking with crossed arms-crossed legs | |
